# Knowledge and attitude towards COVID-19 and associated factors among health care providers in Northwest Ethiopia

**DOI:** 10.1371/journal.pone.0238415

**Published:** 2020-08-28

**Authors:** Belayneh Ayanaw Kassie, Aynishet Adane, Yared Tadesse Tilahun, Eskeziaw Abebe Kassahun, Amare Simegn Ayele, Aysheshim Kassahun Belew

**Affiliations:** 1 Department of Women’s and Family Health, School of Midwifery, College of Medicine and Health Sciences, University of Gondar, Gondar, Ethiopia; 2 Department of Internal Medicine, School of Medicine, College of Medicine and Health Sciences, University of Gondar, Gondar, Ethiopia; 3 EngenderHealth Family Planning by Choice Project, University of Gondar Center of Excellence, Gondar, Ethiopia; 4 Department of Midwifery, College of Health Sciences, Woldia University, Woldia, Ethiopia; 5 Department of Midwifery, College of Medicine and Health sciences, Debretabor University, Debretabor, Ethiopia; 6 Department of Human Nutrition, Institute of Public Health, University of Gondar, Gondar, Ethiopia; Qazvin University of Medical Sciences, ISLAMIC REPUBLIC OF IRAN

## Abstract

**Background:**

COVID-19 has a devastating effect on social, economic, and political crises that will leave deep pockmarks on victims of the virus. Having poor knowledge and attitude of the disease among health care providers could bring in impeded effect in the supportive treatment and, it increases the spread of the pandemic.

**Objective:**

The study aims to assess the knowledge and attitude towards COVID-19, and associated factors among health care providers in Northwest Ethiopia in 2020.

**Methods:**

Institution based cross-sectional study was conducted from the mid of March to the end of April 2020 among 408 participants who were selected by a simple random sampling technique. Pretested and structured self-administered questionnaire was used to collect data. The data were entered using EPI-info v. 7, and were exported to SPSS version 20 for further analysis. Bivariate and multivariable logistic regression analyses were used to identify factors associated with Knowledge and Attitude towards COVID-19. Variables having p-value < 0.05 were taken as variables which were significantly associated with the dependent variable.

**Result:**

A total of 408(97.1%) participants have participated in the study. Most of the participants (67.3%) were males. One-third (35.5%) of the participants were nurses. About 62% of the health care providers were Bachelor degree holders. The prevalence of Knowledge and attitude towards COVID-19 found to be 73.8% (95%CI: 69.9, 77.9) and 65.7% (95%CI: 61.5, 70.1) respectively. Master degree level of education (AOR = 2.85; 95% CI: 1.25, 6.00) was associated with knowledge of the participants. Similarly, having good knowledge (AOR = 3.17; 95%CI: 1.97, 5.06) was positively associated with the attitude of health care providers towards COVID-19.

**Conclusion and recommendation:**

Health care providers found to have good knowledge and attitude towards COVID-19. Being Master’s Degree holder and having good knowledge are associated with the knowledge and attitude of the respondents towards COVID-19 respectively. Thus, improving awareness through health education is a significant approach to address the global agenda of COVID-19 Pandemic.

## Introduction

Coronavirus disease (COVID-19) is a contagious disease caused by a newly discovered coronavirus which was declared as pandemic outbreak since February 11, 2020 [1]. Most people who are infected with COVID-19 virus experience mild to moderate respiratory illness, and they recover without requiring special treatment [1, 2]. Pneumonia like cases of unknown etiology was reported in Wuhan city, China since December 2019, and it was eventually identified as a coronavirus [3].

On January 30, 2020, WHO declared COVID-19 outbreak to be a public health emergency as it continued to dramatically spread across the world; furthermore, the WHO global health emergency committee also stated that the spread of COVID-19 can be reduced by early detection, isolation, prompt treatment, and prompt contact tracing strategies [4]. As of May 21, 2020, more than 5 million people worldwide, 100 thousand people in Africa, and close to 400 people in Ethiopia were confirmed to have been infected with COVID-19 [4].

COVID-19 has socio-economic impacts; Global reports shown that economic growth is lowered by 1, 1.5, and 2.5 percent globally, in Africa and in Ethiopia, respectively [5–7]. This leads Globally, 14–22 million additional people will be living in the extreme poverty [5]. Similarly, 48% of the people are also affected in Africa [8]. Moreover, 0.4–1.2 million additional people are affected by the pandemic, and about half of them are children, and they can go through into poverty [7].

Healthcare providers are on the frontline in the fight against the COVID-19 pandemic while most of the other civil servants are staying at home; as a result, thousands of health care providers are being infected with COVID-19 worldwide while delivering clinical service to COVID-19 patients, and the condition is worse in developing countries with the poor health care system [9]. Reports indicated that, as a result of health care providers’ shortage, and due to the high demand, health care workers are frequently infected with COVID-19. For instance, 20% of health care providers in Italy [10], 26% in Spain [11], 16% in USA [12] and 19.6% in the Netherlands [13] are infected with COVID-19, and the problem will worsen in poor health care settings. In addition, it can increase the incidence of anxiety, mental fatigue, and stress disorder among frontline health care providers [14].

Findings from previous studies revealed that, age [9, 15], news from media [9] were significantly associated with good Knowledge of COVID-19. In spite of the presence of several guidelines, online training sessions, and materials prepared by WHO and others, significant numbers of health care providers remain to have limited knowledge and negative attitude about COVID-19. There is paucity of evidences regarding knowledge and attitude towards COVID-19. To the best of our knowledge, this study is the first to assess the knowledge and attitudes of health care providers towards COVID-19 in Ethiopia. Therefore, this study is intended to assess the current status of the knowledge, attitude, and associated factors towards COVID -19 among health care providers in Northwest Ethiopia.

## Methods

### Study setting, design and period

An Institution based cross-sectional study was conducted from the mid of March to the end of April 2020 in the Central Gondar zone, Northwest Ethiopia, located 166 km from Bahir Dair City the capital of Amhara regional state and 748 km from the capital city of Ethiopia, Addis Ababa. There are 15 Administrative districts, 10 hospitals, 85 health centers, and 433 health posts in the administrative zone. According to the 2019/20 Ethiopian population projection, the central Gondar zone has a total population of about 2,788,442.

### Source and study population

All health care providers who were working in Central Gondar zone health facilities used as the source population. All health care providers working in the selected health facilities from Central Gondar zone were the study population.

### Sample size and sampling procedure

The sample size was estimated by using the single proportion formula using the following assumptions; 50% prevalence, as of the there is no any previous study, confidence interval (CI) 95%, Z as 1.96, and margin of error as 5% and 10% of non-response rate. Finally, 420 was the final sample size considered in the current study. Participants were selected using a multistage sampling technique. Primarily, three hospitals and seven health centers selected using a simple random sampling technique. Then, the total sample proportionally allocated for selected health facilities based on their number of health care providers. Finally, Simple random sampling technique was applied to identify individual participants from selected health facilities.

### Data collection tool and procedures

A pretested and structured self-administered questionnaire was used to collect the data with all necessary precautions for COVID-19 prevention. The tool was adopted and modified in to the local context from previously published articles [16, 17], CDC [18] and WHO guidelines [19]. Four data collectors and two supervisors were participated in data collection. One day training regarding data collection and necessary COVID-19 precautions was given for data collectors. The questions were classified into socio-demographic characteristics, information source, knowledge, and attitude towards COVID-19. The validity of questionnaire was assessed by pretesting with 21(5%) providers from facilities out of the study area. The reliability was assessed by calculating the Cronbach’s alpha showing acceptable internal consistency. During the day of data collection, no personal identifiers collected, and necessary COID-19 preventive measures applied in the entire process. Completeness and accuracy of the collected data were checked on a daily basis.

### Data processing and analysis

Completeness and consistency of the response were checked manually before the data entry. The data were entered into Epi info V.7 and analyzed using SPSS version 20. Tables, figures, and texts were used to summarize descriptive statics of the study. Mean and the standard deviation were used for numerical data. All continuous variables were checked for normality Hosmer-Lemeshow goodness of fit test. Logistic regression was employed to assess the association between dependent and independent variables. Odds ratio (OR) with 95% CI was used to assess the strength of association, and p-value <0.05 for statistical significance of knowledge and attitude.

### Measurement

The providers’ knowledge were assessed with 27 question consisting causative agent, incubation period, possible commonest symptoms, treatment and vaccination availability, modes of transmission and prevention mechanisms. Each question contain three response; ‘Yes’, ‘No’ and ‘I don’t know’ and coded as 1 for correct and 0 for incorrect responses, then after computing all knowledge scores, those respondents who respond above the mean score were considered as having good knowledge, whereas, below the mean value labeled as poor knowledge. The attitude section included 11 questions composed of confidence on COVID-19 control, attitude towards preventive interventions, fear to acquire or transmit the disease and interest to be involved in COVID-19 patient care; and each item contain responses ‘Yes’, ‘No’ and ‘Not sure’ components and then coded as 1 for correct and 0 for incorrect responses. Finally, all attitude scores were computed. Those respondents who respond above the mean score were considered as having positive attitude, whereas, below the mean value labeled as negative attitude. Some questions were reversed to eliminate biases of giving a single similar response in all the items.

### Ethical approval and consent to participate

Ethical clearance was obtained from the Institutional Ethical Review Board of Debre Tabor University. Informed written consent was obtained from each health care provider who is targeting after informing them all the purpose, benefits, risks, the confidentiality of the information, and the voluntary nature of participation in the study. The respondents were notified that they had the right to refuse or stop at any point of the data collection.

## Results

### Socio-demographic characteristics

A total of 408 health care providers with a response rate of 97.1% had participated in this study. The mean age (±SD) of the health care providers was 30.33 (± 6.53) years. Three hundred seventy (90.7%) were from the Amhara ethnic group and 377 (92.4%) were orthodox Christian in religion. Two-third, (67.3%) of the participants were males and 267 (65.4%) were in the age range of 20–30 years. Half of the participants (50.7%) were single in marital status. One hundred fourteen (31.1%) of the health care providers have 6–10 years of work experience. Two hundred eighty-five (69.9%) of the participants have been working in a hospital, 144(35.3%) of the participants were nurses by profession and 253(62%) were bachelor degree holders (Table 1).

**Table 1:** Socio-demographic characteristics of the health care providers in central Gondar zone, Northwest, Ethiopia, 2020.

Variables	Frequency	Percent (%)
**Age**		
20–30	267	65.4
31–40	114	27.6
> = 41	27	6.6
**Years of Experience**		
1–5	220	53.9
6–10	114	31.1
> = 11	27	15
**Type of facility working in**		
Health center	123	30.1
Hospital	285	69.9
**Ethnicity**		
Amhara	370	90.7
Qimant	19	4.7
Oromo	10	2.5
Others	9	2.2
**Religion**		
Orthodox	377	92.4
Muslim	21	5.1
Protestant	10	2.5
**Marital status**		
Married	201	49.3
Single	207	50.7
**Profession**		
Radiology	6	1.5
Anesthesia	13	3.2
Health Officer	15	3.7
Pharmacy	26	6.4
Laboratory	45	11
Physician	61	15
Midwife	98	24
Nurse	144	35.3
**Level of Education**		
Diploma	71	17.4
Bachelor degree	253	62
Master degree	84	20.6

### Source of information

**Fig 1:**
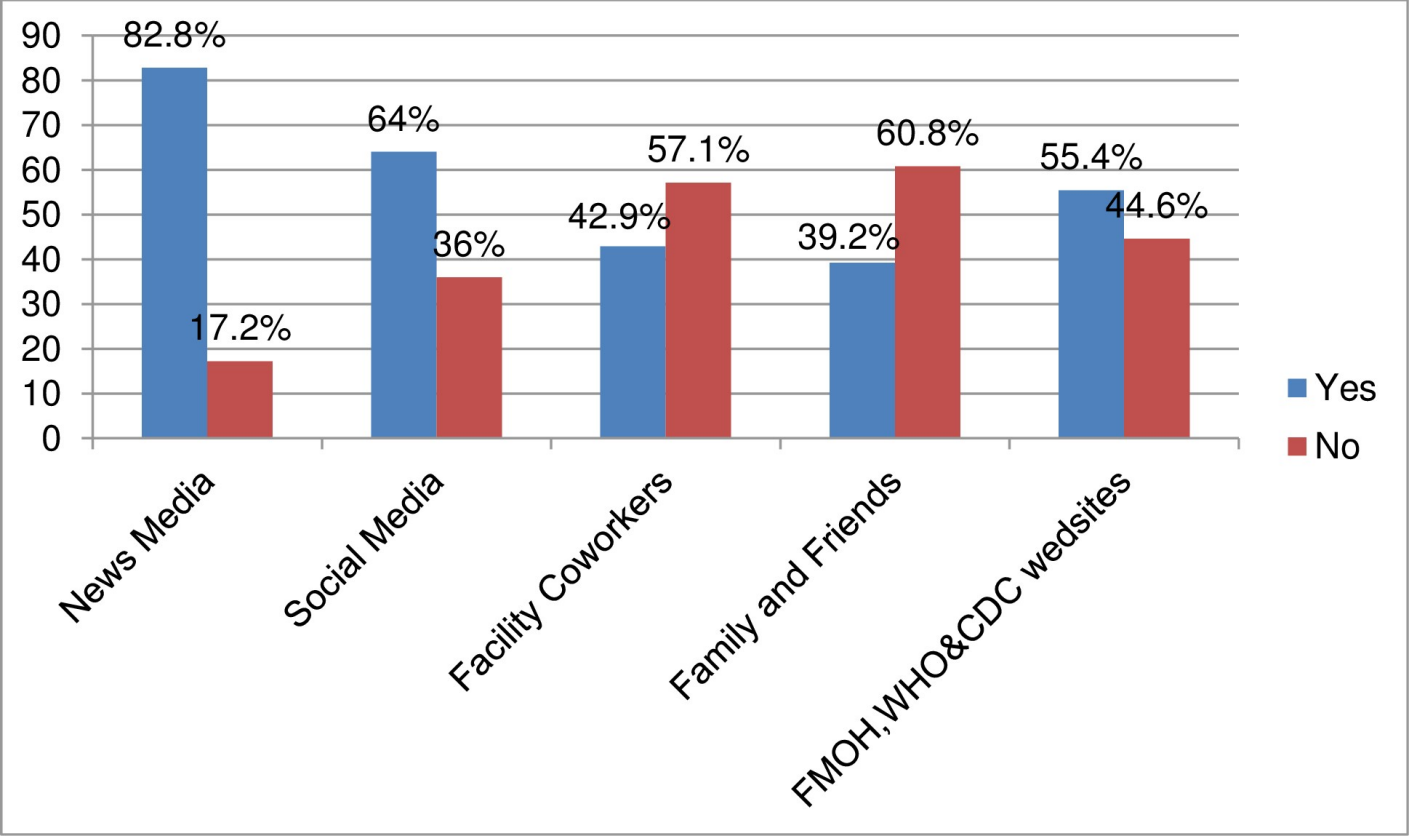
Source of information for health care providers in Central Gondar Zone, Northwest, Ethiopia, 2020.

Almost all 400(98%) of the health care providers had ever heard about COVID-19. Mass media like television and radio was the main source of information for about 82.8% providers. More than half, (64%) of the participants have used websites of the Federal Ministry of Health (FMOH), WHO and Center for Disease Control (CDC) as the source of information (Fig 1).

### Knowledge towards COVID-19

According to the current study, the prevalence of Good Knowledge was found to be 73.8% (95%CI, 69.9–77.9). Three hundred ninety three (96.3%) providers correctly answer as COVID-19 is a viral infection and 94.9% respond the incubation period of COVID-19 infection is 2–14 days. Three hundred eighty-six (94.6%), 348 (85.3%), and 335 (82.1%) professionals mentioned fever, cough, and shortness of breath as commonest symptoms of COVID-19 respectively. Nearly half (45.3%) of the health care providers state mild presentation as the most common form of the severity of presentation in COVID-19 patients. Twenty six (6.4%) of the professionals respond antibiotics are effective in treating COVID-19 infection. Regarding treatment, 85.8% of the professionals respond only symptomatic and supportive care is the current treatment for COVID-19.

### Attitude towards COVID-19

Positive Attitude towards COVID-19 among health care providers found to be 65.7% (95%CI, 61.5–70.1). Among study participants 270(66.2%) think they may probably get infected with COVID-19. Three hundred eighty six (94.6%) and 377(92.4%) believe hand hygiene and wearing face masks are important in controlling the spread of COVID-19, respectively. Among the study participants 361(88.5%) were not interested to be involved in the treatment of COVID-19 patients, and 367 (90%) responds they will not accept isolation in health facilities if they get infected with COVID-19. Three hundred six (75%) believe sick patients should share their recent travel history for their health care providers. Two hundred twenty nine (56.1%) of providers believe the government is not doing enough to prevent and control COVID-19 outbreak, however, 285(69.9%) of the health care providers believe COVID-19 will finally be controlled successfully.

### Factors affecting knowledge towards COVID-19

Both Bivariate and multivariable analyses were applied to assess the effect of dependent variables on knowledge and attitude towards COVID-19. As shown in Tables 2 and 3, after adjusting for confounder variables in the multivariate analyses, having master’s degree and good knowledge towards COVID-19 were significantly associated with good knowledge and positive attitude towards COVID-19 respectively.

**Table 2:** Factors associated with knowledge on COVID-19 among health care providers, Central Gondar Zone, Northwest Ethiopia, 2020.

Variables	Knowledge on COVID-19		COR (95% C)	AOR (95% CI)
	**Poor**	**Good**		
**Sex**				
Male	66(25.4)	194(74.6)	1.40(0.88,2.24)	1.14(0.70,1.85)
Female	41(27.7)	107(72.3)	1	1
**Age**				
20–30	63(23.6)	204(76.4)	1	1
31–40	31(27.2)	83(72.8)	0.83(0.50,1.36)	1.04(0.55,1.97)
> = 41	13(48.1)	14(51.9)	0.33(0.15,0.75)	0.48(0.55,1.43)
**Years of Experience**				
1–5	46(20.9)	174(79.1)	1	1
6–10	38(29.9)	89(70.1)	0.62(0.38,1.02)	0.65(0.36,1.16)
> = 11	23(37.7)	38(62.3)	0.44(0.24,0.81)	0.60(0.23,1.58)
**Types of Facility working in**				
Health center	38(30.9)	85(69.1)	1	1
Hospital	69(24.2)	216(75.8)	1.34(0.88,2.24)	0.95(0.55,1.65)
**Marital status**				
Married	58(28.9)	143(71.1)	0.77(0.49,1.19)	0.88(0.55,1.43)
Single	49(23.7)	158(76.3)	1	1
**Level of Education**				
Diploma	24(33.8)	47(66.2)	1	1
Bachelor degree	71(28.1)	182(71.9)	1.32(0.75,2.30)	1.20(0.66,2.18)
Master degree	12(14.3)	72(85.7)	3.06(1.40,6.71)	2.85(1.25,6.50)*

**Table 3:** Factors associated with attitude on COVID-19 among health care providers, Central Gondar Zone, Northwest Ethiopia, 2020.

Variables	Attitude towards COVID-19		COR (95% CI)	AOR (95% CI)
	**negative**	**Positive**		
**Sex**				
Male	85(32.7)	175(67.3)	1.22((0.80,1.80)	1.13(0.72,1.79)
Female	55(37.2)	93(62.8)	1	1
**Age**				
20–30	89(33.3)	178(66.7)	1	1
31–40	40(35.1)	74(64.9)	0.93(0.58,1.47)	0.95(0.52,1.72)
> = 41	11(40.7)	16(59.3)	0.73(0.32,1.63)	0.93(0.29,3.02)
**Years of Experience**				
1–5	71(32.3)	149(67.3)	1	1
6–10	46(36.2)	81(63.8)	0.84(0.53,1.33)	1.05(0.60,1.84)
> = 11	23(37.7)	38(62.3)	0.79(0.44,1.42)	1.20(0.47,3.05)
**Types of Facility working in**				
Health center	49(39.8)	74(60.2)	1	1
Hospital	91(31.9)	194(68.1)	1.41(0.91,2.19)	1.15(0.68,1.94)
**Marital status**				
Married	78(38.8)	123(61.2)	0.67(0.45,1.02)	0.76(0.49,1.20)
Single	62(30)	145(70)	1	1
**Level of Education**				
Diploma	32(45.1)	39(54.9)	1	1
Bachelor degree	80(31.6)	173(68.4)	1.77(1.04,3.04)	1.62(0.91,2.89)
Master degree	28(33.3)	56(66.7)	1.64(0.86,3.15)	1.24(0.61,2.52)
**Knowledge**				
Poor	58(54.2)	82(27.2)	1	1
Good	49(45.8)	219(72.8)	3.16(2.00,4.99)	3.17(1.97,5.06)*

The odds of having good knowledge towards COVID-19 were 2.85 times higher among Health care providers having master degree [AOR: 2.85; 95% CI (1.25, 6.00)] as compared with health care providers holding diploma(Table 2).

The odds of having positive attitude towards COVID-19 infection were 3.17 times higher among health care providers having good knowledge of COVID-19 [AOR: 3.17; 95%CI (1.97, 5.06)] than providers with poor knowledge towards COVID-19 (Table 3).

## Discussion

Ethiopia is one of the populous countries in East Africa, and the Government of Ethiopia is doing multiple interventions to contain the epidemic before it causes significant damage on the community. The government declared a state of emergency, and it established national COVID-19 response taskforce which inform disease prevention and control directions, mobilize resources to ensure and ration the supplies of personal protective equipment, provide daily situational updates and do massive awareness creation efforts using different social and mass media platforms, and develop different guidelines [20]. As health care providers are frontline targets, they are exposed to COVID-19 while caring for patients [21].

The current study revealed that 98% of the health care providers heard about COVID-19 and 96.3% of them believe that COVID-19 is a global pandemic outbreak that affected the globe. Only 24.8% of the health care providers had attended formal training, discussion and lectures about COVID-19.

This study shows that 73.8% of the health care providers have good knowledge of COVID-19 infection. This finding is lower than the study conducted in China, 88.4% [22]. The possible reason for the difference might be due to the study setting; our study included participants from health centers and hospitals whereas the previous was only hospital based. In addition, health care providers working in hospitals may have the highest skilled manpower as compared to the health centers which are mostly organized with diploma holders, and as of china, where the first COVID-19 outbreak occurred, providers might have better access of information, and are more exposed for cases than in the study of the current study. Moreover, hospitals are also organized with good infrastructures; for instance, the internet and others to capacitate the health care providers towards COVID-19 knowledge.

The study revealed that 65.7% of the health care providers had a positive attitude towards COVID-19. This finding is lower than the study conducted in china which is about 78% [23] had a positive attitude towards the disease. The difference might be due to differences in the country’s setting as China’s health care system, information, protection, and support are so much better than the Ethiopian context which will affect providers’ attitude towards the disease.

Previous studies have shown that during the time of such disease outbreaks, health care workers are prone to mental health diseases [24] and in this study 66.2% of health care providers fear that they may probably get infected with COVID-19, and 90% of the providers respond that they will not accept isolation in isolation centers if they get infected. People under isolation might experience social stigma, get considered as a public health threat, self‑blame, sense of being punished or even discriminated, which may lead to negative consequences on peoples cooperation for disease control [25].

Having a higher educational status is among the factors contributing to better knowledge because of increased opportunity to be exposed to information and knowledge. In this study health care providers having Masters Level of education were found to have good knowledge of COVID-19 than diploma holders. This finding is supported by reports from china [16]. The possible reason might be due to health care providers having higher educational levels could have better opportunities to access local and international information, research, and training platforms than their counterparts [26].

Knowledge is the foundation of everything, and in this study, health care providers who have good knowledge of COVID-19 had found to have a positive attitude towards COVID-19 infection than providers who have poor knowledge of the causes, modes of transmission, and prevention of COVID-19 infection. This is supported by the study in Iran [27] and China [28]. The probable reason might be due to the fact that health care providers having sufficient knowledge towards the route of transmission, characteristics of the disease, diagnosis, treatment, and prevention of COVID-19 will have supported the positive attitude toward COVID-19. Knowing the transmission and prevention of COVID-19 also makes it easier for health care providers to know who and when patients died from COVID-19 because they are aware of the severity of the disease. Moreover, the international and national health sector is playing a key role in reaching and spreading information about the diseases to encourage health care providers in promoting a positive attitude. Furthermore, health education programs by using different media that are intended to improve COVID-19 knowledge which is ready to lend a hand for encouraging a positive attitude [16].

As drugs or vaccines are not yet available, various preventive measures have been recommended to contain the spread of the infection including sanitary measures, case identification, contact tracing, isolation and quarantine, social distancing, travel restrictions and use of personal protective equipments [29, 30]. Though there are diverse level of uncertainty and beliefs regarding the effectiveness of masks [31], 92.4% of the participants believe that wearing face mask is important in controlling the spread of COVID-19. However, due to the sudden rise in the number of cases and the droplet mode of transmission of the infection, the demand for personal protective equipments had surged [32], and shortage of supplies for hand hygiene and lack of PPE have been reported from health facilities [20], which require increasing the rate of production, minimizing the need through promoting rational use, and coordinating the supply chain mechanism [33].

In conclusion, our study indicated that health care providers had good points of knowledge and attitudes towards COVID-19. High educational status and having good knowledge are associated with the knowledge and attitude of the respondents concerning COVID-19. Thus, improving awareness through health education is a significant approach to address the global agenda of COVID-19 Pandemic.

### Limitation of the study

There was no previously standardized and validated tool to assess the Knowledge and attitude of health care providers towards COVID-19. As the area is not well studied, we didn’t find adequate studies to compare and contrast our findings with others, and it makes our discussion shallow. As the study assessed knowledge and attitude, it may not necessarily reflect the actual practice that people comply with. Despite these limitations, our findings provide valuable information about health care providers’ knowledge and attitude towards the disease.

## Supporting information

S1 Data (PDF)

S2 Data (PDF)

## Abbreviations

AOR: Adjusted Odds Ratio
CDC: Center of Disease Control
COVID-19: Coronavirus Disease 2019
CI: Confidence Interval
COR: Crude Odds Ratio
FMOH: Federal Ministry of Health
HCP: Health Care Providers
SPSS: Statistical Package for Social Sciences
WHO: World Health Organization
