# Supplementary material for: Knowledge and attitude towards COVID-19 and associated factors among health care providers in Northwest Ethiopia
Source: PLoS One. 2020 Aug 28;15(8):e0238415. doi: 10.1371/journal.pone.0238415 (PMC7454942; doi:10.1371/journal.pone.0238415)
Supplement: S1 Data — (PDF) [file pone.0238415.s001.pdf]

Information regarding the pretest

Pretest was conducted in order to detect and remediate problems before a standardized tool was finalized among 21 health care providers from two health facilities so as to :

1. To ensure the flow, order, skip patterns, timing, and overall respondent interest and attention
2. To identify the strengths and weaknesses of the survey question, format, wording and variation, and meaning of questions.
3. To check the reliability and validity of the survey questions taken from the World Health Organization (WHO) and similar articles previously published
4. To reduce error, reduce respondent burden, to check whether or not respondents are interpreting questions correctly through:
   - ✓ One day intensive training regarding data collection and necessary precautions was given for data collectors
   - ✓ During the training, make the couple the data collectors for practicing and discussion on the clarity of the question, order and time
   - ✓ After practicing in-group we sent the data collector to conduct pretest in the health facilities
   - ✓ By the next day, we have summarized language and content related comments, procedure to follow, experiences, and challenges found during the pretest
   - ✓ Finally, we took a lesson and reached an agreement about how to ask and help the respondents to answer the question easily by making questions as short and easily understandable as possible, and set clear instructions and direction for each question.
