# Supplementary material for: Knowledge and attitude towards COVID-19 and associated factors among health care providers in Northwest Ethiopia
Source: PLoS One. 2020 Aug 28;15(8):e0238415. doi: 10.1371/journal.pone.0238415 (PMC7454942; doi:10.1371/journal.pone.0238415)
Supplement: S2 Data — (PDF) [file pone.0238415.s002.pdf]

Knowledge and attitude towards COVID-19 and associated factors among health care providers in Northwest Ethiopia

Part -I Question to assess Socio-economic information

Instructions: Write your response on the space provided for open ended questions and Encircle your Response for close ended questions.

S.No.	Questions	Options
101	Type of Health facility you are working?	1. Hospital<br>2. Health center
102	Age	_______________years
103	Sex	1. Male<br>2. Female
104	Profession	1. Physician<br>2. Nurse<br>3. Midwife<br>4. Health officer<br>5. Pharmacy<br>6. Laboratory<br>7. Other(specify)----
105	Level of Education	1. Diploma<br>2. Bachelor Degree<br>3. Masters Degree<br>4. Other ____________
106	Years of experience	_____________years
107	Marital status	1. Single<br>2. Married<br>3. Divorced<br>4. Separate<br>5. Widowed
108	Ethnicity	1. Amahra<br>2. Oromo<br>3. Qimant<br>4. Tigray<br>5. Other (Specify)…..
109	Religion	1. Orthodox<br>2. Muslim<br>3. Protestant<br>4. Other (specify)……….

Part II: Questionnaire of knowledge, and attitudes towards COVID-19

	**Section II. Knowledge, please respond to the following questions**	
S.no	Questions	Options
201	Did you ever heard about COVID-19	1. Yes<br>2. No
202	What was your source of information about Covid-19? (more than one answer is possible)	1. News media (TV,Radio)<br>2. Social media (FB, Telegram etc)<br>3. From facility coworkers<br>4. Family and friends<br>5. Websites of FMOH,WHO,CDC etc<br>6. Other specify………
203	Covid-19 is a viral infection	7. Yes<br>8. No<br>9. Don't know
204	The incubation period of Covid-19 is?	1. 24 hours<br>2. 1-5 days<br>3. 2-14 days<br>4. Don't know
205	How long is the quarantine period for COVID-19 suspect?	1. 1 week<br>2. 2 weeks<br>3. 3 weeks<br>4. weeks
206	Asymptomatic persons with COVID-19 can transmit the disease to other people	1. Yes<br>2. No<br>3. 3. I don't know
207	Health care workers are at higher risk of infection?	1. Yes<br>2. No<br>3. Don't know
208	Which group of population can be infected by COVID-19?	1. Adults<br>2. Children<br>3. Elderly<br>4. All can be infected
209	COVID-19 has an air born transmission?	1. Ye<br>2. No.<br>3. Not clearly defined<br>4. I don't know
210	Covid-19 is transmitted via respiratory droplets?	1. Yes

		2. No<br>3. Don’t know
211	The following practices can help to protect you from COVID-19?<br>(check all that can apply)	1. Washing your hands with soap/use hand sanitizers frequently<br>2. Covering your nose with your hands during sneezing<br>3. Wearing a face mask<br>4. Keeping physical distance<br>5. Staying at home/avoid crowds<br>6. Other specify……………….
212	What are the possible typical symptoms of Covid-19? (check all that can apply)	1. Fever<br>2. Dry cough<br>3. Sore throats<br>4. Shortness of breath<br>5. fatigue<br>6. myalgia<br>7. Others specify ………
213	How do you confirm diagnosis of COVID-19 by?	1. Nasopharyngeal/Throat swab<br>2. CXR<br>3. Blood sample<br>4. Stool sample
214	Most common form of severity of presentation for COVID -19 patients is?	1. Mild<br>2. Severe<br>3. Critical
215	COVID -19 could be fatal	1. Yes<br>2. No<br>3. Don’t know
216	Risk of infection and death from COVID-19 is higher among patients with underlying chronic illness	1. Yes<br>2. No<br>3. I don’t know
217	Are there specific vaccines to prevent Coronavirus disease (COVID-19)?	1. Yes<br>2. No<br>3. I don’t know
218	Are antibiotics effective in preventing and treating Corona virus disease (COVID-19) ?	1. Yes<br>2. No<br>3. I don’t know

219	Are there specific medicines to treat Corona virus disease (COVID-19) as of today?	1. Yes<br>2. No<br>3. Not sure
220	Symptomatic and supportive care is the current treatment for covid -19?	1. Yes<br>2. No<br>3. I don't know
	**Section III. Attitudes, please respond to the following questions**	
301	Do you believe that COVID-19 will finally be successfully controlled?	1. Yes<br>2. No<br>3. I don't know
302	Do you think Hand Hygiene is important in controlling the spread COVID-19	1. Yes<br>2. No<br>3. I don't know
303	Do you think wearing masks is important in controlling the spread covid -19	1. Yes<br>2. No<br>3. I don't know
304	Do you think you may probably get infected with covid -19?	1. Yes<br>2. No<br>3. No sure
305	If you get infected with Covid -19 will you accept isolation in health facilities?	1. Yes<br>2. No
306	Do you fear that you may transmit COVID-19 to your family members?	1. Yes<br>2. No
307	Do you think that every health care worker working at a COVID-19 treatment center need to be quarantined though asymptomatic?	1. Yes<br>2. No
308	Are you interested to be involved in the treatment of Covid-19 patients?	1. Yes<br>2. No
309	Sick patients should share their recent travel history for their health care providers?	1. Yes<br>2. No
310	Do you have confidence that Ethiopia can win the battle against the COVID-19 virus?	1. Yes<br>2. No<br>3. I don't know
311	Do you think the government of Ethiopia is doing enough to prevent and control Corona virus disease (COVID-19) outbreak?	1. Yes<br>2. No<br>3. Not sure
